# Supplementary material for: Chlamydia interfere with an interaction between the mannose-6-phosphate receptor and sorting nexins to counteract host restriction
Source: eLife. 2017 Mar 2;6:e22709. doi: 10.7554/eLife.22709 (PMC5364026; doi:10.7554/eLife.22709)
Supplement: Supplementary file 3. — DOI: http://dx.doi.org/10.7554/eLife.22709.016 [file elife-22709-supp3.doc]

**Supplementary File 3.** Data collection and refinement statistics

|  | SNX5-PX:IncE |
| --- | --- |
| **Data collection** |  |
| X-ray source | ALS 8.3.1 |
| X-ray wavelength (Å) | 1.11587 |
| Space group | P 2_1_ |
| Cell dimensions |  |
| *a, b, c* (Å) | 36.73, 110.10, 86.13 |
| β (°) | 90.34 |
| Resolution (Å) ^a^ | 67.84-2.31 (2.43-2.31) |
| No. of unique reflections | 29,845 (4,320) |
| R_meas_ (%) | 19.9 (149.8) |
| Mean I/σ(I) | 6.5 (1.3) |
| CC_1/2_ | 0.987 (0.332) |
| Completeness (%) | 99.4 (98.7) |
| Redundancy | 3.6 (3.6) |
| **Refinement** |  |
| Resolution (Å) | 67.84-2.31 |
| No. of reflections | 29,799 |
| R_work_/R_free_ (%) | 20.32/26.00 |
| No. of atoms |  |
| Protein | 5,549 |
| Water | 252 |
| Average B-factors (Å^2^) |  |
| SNX5-PX, chain A | 43.15 |
| IncE, chain P | 53.89 |
| Water | 39.65 |
| R.m.s. deviations |  |
| Bond lengths (Å) | 0.004 |
| Bond angles (°) | 0.640 |
| Ramachandran plot (%) ^b^ |  |
| Favored regions | 97.19 |
| Allowed regions | 2.81 |
| PDB ID | 5TP1 |

^a^ Values in parentheses refer to the highest resolution shell.

^b^ As given by MolProbity ***(Chen et al., 20***10).

**Chen,** VB, Arendall, WB, 3rd, Headd, JJ, Keedy, DA, Immormino, RM, Kapral, GJ, Murray, LW, Richardson, JS, and Richardson, DC. 2010. MolProbity: all-atom structure validation for macromolecular crystallography*. Acta Crystallographica Section D Biological Crystallograph***y** 66:12-21.
