# Supplementary material for: Chlamydia interfere with an interaction between the mannose-6-phosphate receptor and sorting nexins to counteract host restriction
Source: eLife. 2017 Mar 2;6:e22709. doi: 10.7554/eLife.22709 (PMC5364026; doi:10.7554/eLife.22709)
Supplement: Supplementary file 4. — DOI: http://dx.doi.org/10.7554/eLife.22709.017 [file elife-22709-supp4.doc]

| **Name** | **Primer** | **Construct** |
| --- | --- | --- |
| mSNX5 FOR 20 | CAAGGACCGAGCAGCCCCTCCTCTGTGTCTGTGGACCTGAATG | pH3C-LIC/SNX5 PX (AA20-180) |
| mSNX5 REV 180 | ACCACGGGGAACCAACCCTTATTACTTGGTATTCTTCCGTCTAACACTTAG | pH3C-LIC/SNX5 PX (AA20-180) |
| mSNX5_t394g_antisense | TCTTAAAGACAGCGAGATCCTCAGCTTCCAGTTCTTG | pFLAG-SNX5 FL (Y132D) and pFLAG-SNX5 FL (Y132D, F136D) |
| mSNX5_t394g_FOR | CAAGAACTGGAAGCTGAGGATCTCGCTGTCTTTAAGA | pFLAG-SNX5 FL (Y132D) |
| mSNX5_t394a_antisense | TCTTAAAGACAGCGAGATTCTCAGCTTCCAGTTCTTG | pFLAG-SNX5 FL (Y132N) and pFLAG-SNX5 FL (Y132N, F136N) |
| mSNX5_t394a | CAAGAACTGGAAGCTGAGAATCTCGCTGTCTTTAAGA | pFLAG-SNX5 FL (Y132N) |
| mSNX5_t406g_t407a | AGCTGAGTATCTCGCTGTCGATAAGAAGACTGTGTCCACC | pFLAG-SNX5 FL (F136D) |
| mSNX5_t406g_t407a_antisense | GGTGGACACAGTCTTCTTATCGACAGCGAGATACTCAGCT | pFLAG-SNX5 FL (F136D) |
| mSNX5_t406a_t407a_antisense | GGTGGACACAGTCTTCTTATTGACAGCGAGATACTCAGCT | pFLAG-SNX5 FL (F136N) |
| mSNX5_t406a_t407a | AGCTGAGTATCTCGCTGTCAATAAGAAGACTGTGTCCACC | pFLAG-SNX5 FL (F136N) |
| IncE LIC FOR 101 | CAAGGACCGAGCAGCCCCTCCAAGTTGCCATGCAAATCGAGTCCAG | pHM3C-LIC/*C. trachomatis* IncE (AA101-132) N-terminal 6xHis-MBP |
| IncE LIC REV 132 | ACCACGGGGAACCAACCCTTATTATTAAACGGGCCCCTTCTCGAACTG | pHM3C-LIC/*C. trachomatis* IncE (AA101-132) N-terminal 6xHis-MBP |
| Infusion_1 | TACCGAGCTCGGATCCCTGGTTTAGTGAACCGTCAGATCCGCT | pcDNA4.0/*C. trachomatis* IncE (AA101-132) C-terminal 2xStrepII |
| Infusion_2 | CTCCCTCGAGCGGCCGCGGTACCGTCGACTGCAGAATTCGAAGCTT | pcDNA4.0/*C. trachomatis* IncE (AA101-132) C-terminal 2xStrepII |

**Supplementary File 4**. List of primers
